# Supplementary material for: Intestinal parasitic infection among household contacts of primary cases, a comparative cross-sectional study
Source: PLoS One. 2019 Oct 7;14(10):e0221190. doi: 10.1371/journal.pone.0221190 (PMC6779256; doi:10.1371/journal.pone.0221190)
Supplement: S1 Questionnaire — (DOCX) [file pone.0221190.s001.docx]

**Consent Form for Participation in a Research Study**

**Title of Study: *Household contact screening of intestinal parasites***

**Description of the research**

You are invited to participate in a research study conducted by the university of Bahir dar CMHS staff. The purpose of this research is to know the burden of intestinal parasitic infection among household contact of known cases.

**Risks and discomforts**

There are no known risks associated with this research.

**Potential benefits**

Patients with anemia or intestinal parasitic infection will be diagnosed using standardized procedures and advised intervention mechanism to avert their risk. This research gives important clue in averting intestinal parasitic infection in the community.

**Protection of confidentiality**

The confidentiality of the data will be kept at all stages.

**Voluntary participation**

Your participation in this research study is voluntary. You may choose not to participate and you may withdraw your consent to participate at any time. You will not be penalized in any way should you decide not to participate or to withdraw from this study.

**Contact information**

If you have any questions or concerns about this study or if any problems arise please contact us using 0918312095/0930111853/0912332655/0918705245.

**Consent**

**I have read this consent form and have been given the opportunity to ask questions. I give my consent to participate in this study.**

Participant’s signature_______________________________ Date:_________________

A copy of this consent form should be given to you.

**Questions**

1. name of the kebele _________
2. average rainfall of the kebele _______________
3. average temperature of the kebele ______________
4. name of the got __________
5. list house hold members

| ***SN*** | ***Relationship with the household head*** | ***Sex*** |
| --- | --- | --- |
|  |  |  |
|  |  |  |
|  |  |  |
|  |  |  |
|  |  |  |
|  |  |  |
|  |  |  |

1. age in years ___________
2. sex ___________
3. occupation ______________
4. residence
5. urban
6. rural
7. marital status
8. single
9. married
10. divorced
11. widowed
12. educational status
13. illiterate
14. read and write
15. formal education
16. family size ______
17. role in the household
18. household head
19. child
20. mother
21. father
22. son in law
23. grand son
24. other relatives
25. average household monthly income________
26. does the household contain latrine
27. yes
28. no
29. the type of latrine
30. traditional latrine
31. modern latrine
32. the sanitation of the house compound
33. Very clean
34. Few waste materials in the compound
35. Dirty environments
36. Did you washed your hand (more than one answer possible)
37. Before feeding yourself/child
38. After visiting the toilet
39. Before cooking your food
40. I didn’t have hand washing practices
41. On average frequency of taking body shower in a week _________
42. The clothes of the respondent during the interview
43. Very clean
44. Clean
45. Not clean
46. The finger nail of the respondents
47. Short
48. Not short
49. The fingernails of the respondents
50. Clean not clean
51. Clean
52. Does the respondents have a habit of walking bare foot
53. Yes
54. No
55. Lighting of the house
56. Electricity
57. Solar
58. Traditional
59. Does the respondents have a habit of ingesting raw vegetables
60. Yes
61. No
62. Habit of playing with domestic animals in the house
63. Yes
64. No
65. Height in cm ______
66. Weight in KG-------------
67. MUAC _________
68. Did the respondent have known chronic illness?
69. Yes
70. No
71. If yes please mention the disease ___________________
72. Water source of the house
73. Piped water
74. Wall water
75. River
76. Pond
77. Other please mention ___________
78. Does the house contain the following things (more than one answer possible)
79. Windows
80. Chimney
81. Room to individual ratio of >1.5
82. Floor made from cement
83. Average time needed to reach to the nearest health facility _____________
84. Is there public waste collection bin around the village?
85. Yes
86. No
87. Does the house contain water filtering materials
88. Yes
89. No
90. Average distance from water source ______________________
91. Does the house contains chicken
92. Yes
93. No
94. Index Stool examination results _____________________________________________________________________________________________________________________________________________________________________________________________________________________________________________.
95. Index Intensity of infection _______________________________
96. Contact stool examination results _____________________________________________________________________________________________________________________________________________________________________________________________________________________________________________.
97. Contact intensity of infection _______________________________
98. Index hemoglobin concentration _______________
99. Index mean corpuscular volume ______________________________________________________
100. Index mean hematocrite concentration _____________________________________________________________
101. Index mean corpuscular hemoglobin
102. Index other RBC indices

____________________________________________________________________________________________________________________________________________________________________________________________________________

1. Contact hemoglobin concentration _______________
2. Contact mean corpuscular volume ______________________________________________________
3. Contact mean hematocrite concentration _____________________________________________________________
4. Contact mean corpuscular hemoglobin
5. Contact other RBC indices
