# Supplementary material for: Intestinal parasitic infection among household contacts of primary cases, a comparative cross-sectional study
Source: PLoS One. 2019 Oct 7;14(10):e0221190. doi: 10.1371/journal.pone.0221190 (PMC6779256; doi:10.1371/journal.pone.0221190)
Supplement: Attachment — Submitted filename: Response to the reviewers.docx [file pone.0221190.s003.docx]

**Response to the reviewers**

Although the authors have answered the reviewer's questions appropriately, however, minor errors should be amended before its suitable publication.

1.Table 2: intestinal parasites—the parasite name should be e.g., Ascaris lumbricoides instead Ascaris Lumbricoides. The first letter of the species should be lowercase.

- Response: revised based on the comment

This results was in line with finding from Sudan and central African republic [20, 21], higher than finding from Uganda [22], and England [23].
Please show their prevalence, respectively. Also, please check the grammar and spellings.

- Response: revised based on the comments.
